# Hem-o-lok Clips Migration: An Easily Neglected Complication after Laparoscopic Biliary Surgery

**DOI:** 10.1155/2017/7279129

**Published:** 2017-09-14

**Authors:** Jun-wen Qu, Gui-yang Wang, Zhi-qing Yuan, Ke-wei Li

**Affiliations:** Department of Biliary-Pancreatic Surgery, Renji Hospital, School of Medicine, Shanghai Jiao Tong University, No. 160, Pujian Road, Pudong New Area, Shanghai 200127, China

## Abstract

Clip migration into the common bile duct (CBD) is a rare but well-established phenomenon of laparoscopic biliary surgery. The mechanism and exact incidence of clip migration are both poorly understood. Clip migration into the common bile duct can cause recurrent cholangitis and serve as a nidus for stone formation. We present a case, a 54-year-old woman, of clip-induced cholangitis resulting from surgical clip migration 12 months after laparoscopic cholecystectomy and laparoscopic common bile duct exploration (LC+LCBDE) with primary closure.

## 1. Introduction

Laparoscopic cholecystectomy and laparoscopic common bile duct exploration (LC+LCBDE) is currently a widely use technology for patients with gallstone and choledocholithiasis. Clip migration into the common bile duct (CBD) is a rare complication of laparoscopic biliary surgery. Surgical clip migration into the common bile duct can cause recurrent cholangitis and serve as a nidus for stone formation. Up to date, few cases of surgical clip migration have been reported in the literature. The etiology and exact incidence of clip migration are both unclear. We report a case of Hem-o-lok clips migration 1 year after laparoscopic cholecystectomy and laparoscopic common bile duct exploration (LC+LCBDE) with primary closure. The patient was successfully treated with endoscopic sphincterotomy plus balloon dilation (ESBD) and Hem-o-lok clips extraction. The patient improved uneventfully following the procedure. We hope that this case draws laparoscopic surgeon's attention to this rare phenomenon.

## 2. Case Presentation

A 54-year-old woman had undergone a successful LC+LCBDE in our hospital on December, 2015. The operation had been performed without difficulty and we had used 2 Hem-o-lok for the duct/artery. Subsequently, primary closure of the incision of CBD was performed with an interrupted 5-0 absorbable suture. A bile leakage was detected on postoperative day 2 and spontaneously resolved after 18 days of conservative treatment. The patient was discharged uneventfully on postoperative day 20.

**Figure 1:**
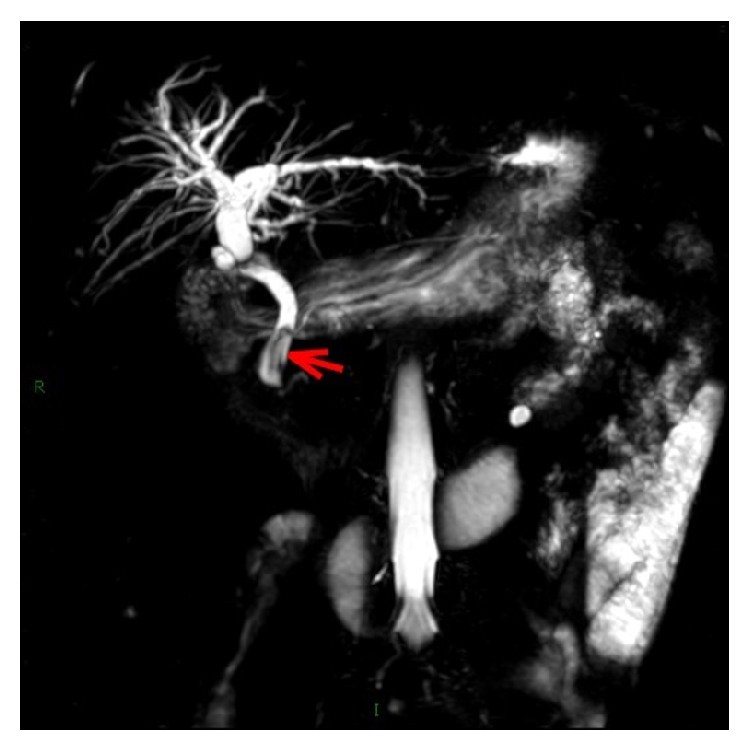
Magnetic resonance cholangiopancreatography (MRCP) showed a slightly dilated common bile duct with a low signal filling-defect in the distal common bile duct, considering CBD stone, with the filling-defect being represented by a red arrow.

**Figure 2:**
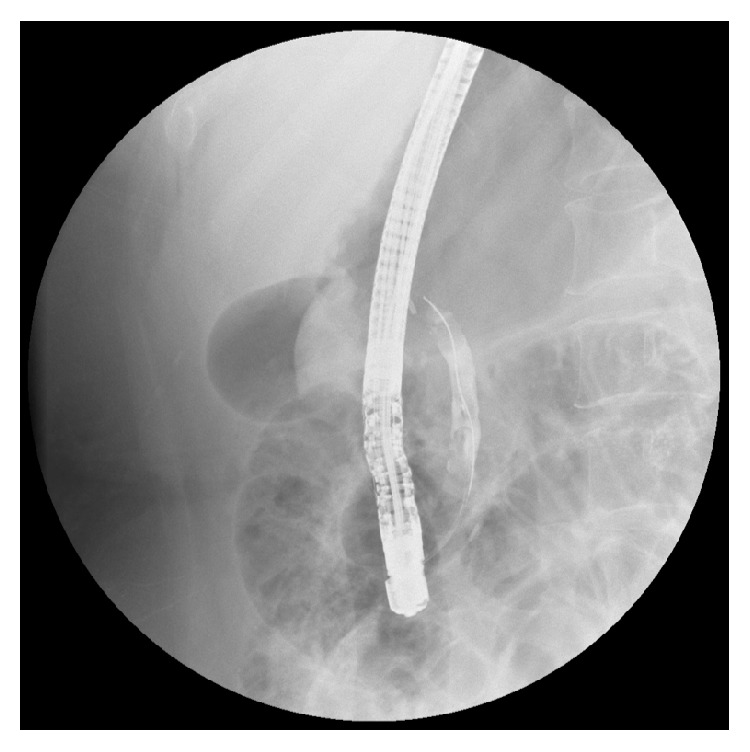
Endoscopic retrograde cholangiopancreatography demonstrates the filling-defect in the common bile duct.

**Figure 3:**
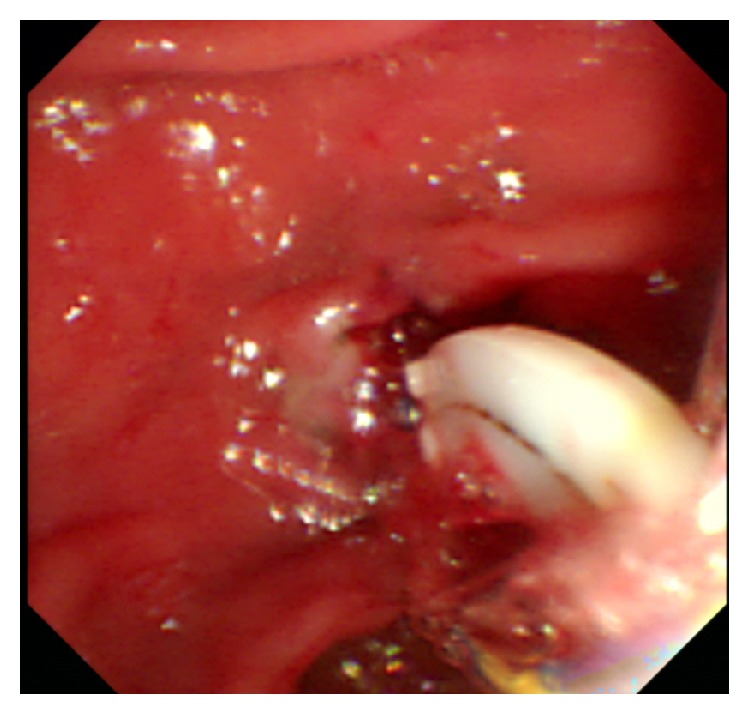
The extracted Hem-o-lok clip within the duodenal lumen.

On January, 2017, this patient was readmitted for intermittent upper abdominal pain for a month without fever and jaundice. Physical examination revealed tenderness in the right upper quadrant of her abdomen. Laboratory examination showed no abnormal parameters. Abdominal US revealed a mildly dilated biliary tree with no visualized CBD stone. Magnetic resonance cholangiopancreatography (MRCP) showed a slightly dilated common bile duct with a low signal filling-defect in the distal common bile duct, considering CBD stone (Figure 1). During ERCP, only a single filling-defect was seen in the common bile duct (Figure 2). An endoscopic sphincterotomy with balloon dilation was carried out and two Hem-o-lok clips were successfully removed from the bile duct with an extraction balloon (Figure 3). An endoscopic nasobiliary drainage (ENBD) tube was inserted after extraction. At one-month follow-up the patient was symptom-free.

## 3. Discussion

Advances in laparoscopy have made LC+LCBDE a widely accepted strategy for patients with gallstones and choledocholithiasis. The single-stage surgical strategy has been shown to be safe, effective, and cost-effective with shorter hospital stays [1]. Exposure of Calot's triangle and securing the gallbladder vessels and cystic duct are the key steps of the LC. Currently, Hem-o-lok clip is widely applied in the controlling of the cystic duct and artery.

Postoperative clip migration is a rare but well-established complication. Migration into the common bile duct after laparoscopic cholecystectomy was first reported in 1992 [2]. Although a huge number of LC have been performed worldwide up to 2016, less than 100 cases of clip migration after LC have been reported in the literature. Most of the cases are metal clips migration. Clip migration tends to develop from 11 days to 20 years; the median time was 26 months [3]. Apart from choledocholithiasis, clip migration may also lead to acute pancreatitis [4], duodenal ulcer [5], biliary-colonic fistula [6], subdiaphragmatic abscess [7], and so forth. To our knowledge, Hem-o-lok clips migration into CBD after LC+LCBDE with primary closure has not been previously reported.

However, the exact mechanism of this condition remains controversial. Multifactor may contribute to the process: the first possible pathogenesis is inappropriate application of clips including incomplete closure of cyst duct and incorrect placement of clips that result in biloma. The number of endoclips used during the initial operation is also an important factor [3, 8]. The second possible pathogenesis is bile leakage caused by intraoperative bile tract injury. In the present case, a primary closure of CBD was performed after LC+LCBDE. Bile leakage was detected after the surgery, and the subhepatic suction drained biliary fluid until it stopped spontaneously on postoperative day 18. We postulate that the subsequent adhesion and inflammation caused by bile leakage make surrounding tissues brittle and induced Hem-o-lok clips detachment and migration from the cystic duct/artery stump into the biliary tract through the incision of CBD mechanically. According to Rawal, the pressure exerted from intra-abdominal organ movements accelerates the process of clip migration [9]. Rejection response of human body to surgical clips may also contribute to the process.

To prevent the incidence of clip migration, all the technical factors in the surgery should be considered: confirming the relationship of Calot's triangle during dissection, minimizing the number of clips, and avoiding unnecessary surgical procedures [10]. The placement of clips should not be too close to the common bile duct. Absorbable clips seem to be a safe and effective method in providing hemostasis in the cystic artery and during ligation of the cystic duct [11]. In three studies, application of absorbable clips brings fewer complications compared to nonabsorbable clips [12–14]. Absorbable clips can burden a higher weight and intraluminal pressure. Furthermore, the biocompatible and nonallergic materials of absorbable clips seldom cause rejection response [11]. Absorbable sutures are also used to seal the cystic artery and duct in some studies [15, 16]. However, the knot is made totally intracorporeally that should be done by an experience surgeon. In spite of many advantages, Francesco Cetta indicated that absorbable clips and sutures are still a nidus for subsequent stones formation [10]. Besides, clipless cholecystectomy using ultrasound dissection technology is an alternative to standard clip of cystic duct and artery. Kandil and Kavlakoglu reported that harmonic scalpel has been proved to be as effective and safe as surgical clips in the ligation of the cystic duct after a period of training [17, 18]. Nonetheless, harmonic scalpel is an expensive device. From the health economics, the clinical application of harmonic scalpel cannot be routinely carried out currently. Primary closure of the CBD has been demonstrated to be a safe and feasible method with a similar rate of bile leaks and recurrent stones compared with that of the T-tube insertion [19]. Advanced laparoscopic skills and experience and appropriate patient selection are the main limitations of LCBDE with primary suture. In our view, further studies are needed to establish more effective and safe methods to prevent complications after LC and LCBDE.

Clinical manifestations of clip migration-induced cholangitis are similar to those of noniatrogenic ones, usually present with abdominal pain, fever, and obstructive jaundice. In our case, pure abdominal pain made us consider the postcholecystectomy syndrome at first. Thus, it emphasizes the importance of early recognition of this complication when patients present with upper abdominal pain or symptoms of biliary obstruction after LC. The diagnosis is suspected on the basis of noninvasive imaging. Ultrasonography is cheap, widely available, and safe and is recommended for patients with suspected foreign body. CT scan is widely used to investigate patients with pain or other abdominal symptoms. MRCP provides clear anatomy of biliary tree with a high sensitivity and specificity.

ERCP is a superior approach to manage the complication with a high success rate of about 85% [3]. In our case, limited endoscopic sphincterotomy with balloon dilation (ESBD) was performed to extract the Hem-o-lok clips. ESBD is a promising technique in treating common bile duct stones with a high success rate of stone extraction and a low risk of complications. Surgical procedures or percutaneous transhepatic cholangiography should be reserved as rescue procedures when ERCP fails [20]. In addition, spontaneous passage of clips through sphincter of Oddi after a failed ERCP extraction or LC had been also reported in some literatures [21, 22].

In conclusion, postoperative clip migration has been a well-recognized phenomenon ever since their first use in surgery, albeit rare. Patients who had LC and LCBDE should have a careful surveillance and strict follow-up to ensure the safety. The first investigation might be MRCP and then ERCP for subsequent management.
